# Design of Materials for Bone Tissue Scaffolds

**DOI:** 10.3390/ma14205985

**Published:** 2021-10-12

**Authors:** Antonio Boccaccio

**Affiliations:** Dipartimento di Meccanica, Matematica e Management, Politecnico di Bari, 70125 Bari, Italy; antonio.boccaccio@poliba.it; Tel.: +39-080-5963393

**Keywords:** bone tissue engineering, porous materials, bone regeneration

## Abstract

The strong impulse recently experienced by the manufacturing technologies as well as the development of innovative biocompatible materials has allowed the fabrication of high-performing scaffolds for bone tissue engineering. The design process of materials for bone tissue scaffolds represents, nowadays, an issue of crucial importance and the object of study of many researchers throughout the world. A number of studies have been conducted, aimed at identifying the optimal material, geometry, and surface that the scaffold must possess to stimulate the formation of the largest amounts of bone in the shortest time possible. This book presents a collection of 10 research articles and 2 review papers describing numerical and experimental design techniques definitively aimed at improving the scaffold performance, shortening the healing time, and increasing the success rate of the scaffold implantation process.

Scaffolds for bone tissue engineering are porous materials that are used to reconstruct large dimensions bone defects. The ideal scaffold should satisfy to the following three principal requirements: (1) it should exhibit a structural response that is adequate and as close as possible to that of the tissues adjacent to the fracture site; (2) it should be biocompatible and biodegradable; (3) it should possess adequate surfaces capable of promoting the adhesion of mesenchymal stem cells, their proliferation and their subsequent osteogenic differentiation [1]. It is commonly known that the rate of bone tissue regeneration and the cellular response is significantly influenced by: (a) the scaffold mechanical behavior, which is, in turn, a function of the scaffold micro-architecture and of the mechanical properties of the material it is made from [2,3]; (b) the surface roughness status and the biological/chemical response of the scaffold/tissue interface surfaces to external factors [4]. The adhesion of stem cells to the scaffold surface as well as the tissue differentiation process occurring in the scaffold pores are regulated by very complex mechanobiological mechanisms taking place at both the micro- (i.e., some micrometers, approximately the dimension of a stem cell) and macro- (i.e., some hundreds of micrometers, corresponding to the typical dimensions of scaffold pores) levels, respectively [5,6,7,8,9]. The scaffold surface must be adequately structured to favor the adhesion of stem cells and their consequent differentiation. Similarly, the scaffold architecture must be properly shaped, and the scaffold material must be adequately designed to trigger favorable biophysical stimuli, leading to the formation of the bony tissue.

Many studies have recently been conducted to investigate the optimal manufacturing technologies that can be used to fabricate “smart and custom” scaffolds capable not only of guaranteeing the above-mentioned requirements, but also of satisfying the specific requests of the specific patient in whom it will be implanted [5]. One of the most recent research lines, in fact, has been focused on the design of “personalized” scaffolds that better suit the anthropometric features of the patient, thus allowing to achieve a successful follow-up in the shortest possible time [10]. Different studies have recently been published with the aim of better understanding the relationship between the scaffold geometry/material properties and the consequent mechanobiological phenomena taking place inside the scaffold during the regeneration process. However, no clear explanations are yet available on the relationship existing between the mechanical/chemical environment and the consequent biological response of tissues occupying the scaffold pores. This Special Issue attempts to bridge the gap and to give a possible response to the open questions.

Most of the studies of the Special Issue developed innovative materials favoring the formation of new bone in the fracture site where the scaffold is implanted [11,12,13,14,15,16]. Three papers investigate the issues related to the geometry/dimensions that the scaffold pores must possess to guarantee an adequate mechanobiological response [10,17,18]. Finally, three articles deal with more clinical/applicative aspects [19,20,21].

The studies investigating innovative materials concern not only the material the scaffold is made from, but also all the materials in presence of which mesenchymal stem cells can be put to favor their adhesion, proliferation, and differentiation. In detail, Nicoara et al. [12], synthesized and characterized two types of materials—with antibacterial properties provided by silver nanoparticles (AgNPs)—based on hydroxyapatite and bacterial cellulose, that are known to possess excellent biocompatibility and bioactivity properties and are, hence, particularly suited to be used in the field of bone tissue engineering. The obtained composite materials were found to have a homogenous porous structure, a high water absorption capacity, and a considerable antimicrobial effect due to silver nanoparticles embedded in the polymer matrix. The fabrication of a composite bone cement made of graphene oxide and poly(methyl methacrylate) was described by Krukiewicz et al. [14], who investigated the potential of this cement to enhance the osteogenic differentiation of human primary mesenchymal stem and progenitor cells. Bastos et al. [15] developed an advanced three-dimensional (3D) biomaterial by integrating bioactive factors, such as lactoferrin and hydroxyapatite, within gellan gum spongy-like hydrogels. The authors demonstrated that that gellan gum spongy-like hydrogels gathered favorable 3D bone-like microenvironment with an increased human adipose-derived stem cells viability. Ishida et al. [16], evaluated starfish-derived β-tricalcium phosphate obtained by phosphatization of starfish-bone-derived porous calcium carbonate as a potential bone substitute material. They concluded that starfish-derived β-tricalcium phosphate may be effective for bone regeneration applications, such as in the treatment of fractures and bone loss. The osteoblastic features of adult mesenchymal stem cells integrated with 3D-printed polycarbonate scaffolds differentiated in the presence of oligostilbenes, such as resveratrol and polydatin, were investigated by Posa et al. [13]. They found that both resveratrol and polydatine stimulate the adhesion of the mesenchymal stem cells to the bone matrix protein osteopontin via α_V_β_3_ integrin and, specifically, polydatine treatment prompted a greater reorganization of this integrin in focal adhesion sites. The effects of a titanium surface coated with polylysine homopolymers on the cell growth of dental pulp stem cells and keratinocytes was investigated by Contaldo et al. [11]. They found an increase in cell growth for both cellular types cultured with polylysine-coated titanium compared to cultures without titanium and those without coating.

Very interesting are also the studies investigating the geometry of the scaffold pores, as well as the issues related to the structural response to mechanical loads and the scaffold porosity. Percoco et al. [17] and Rodríguez-Montaño et al. [10], using the mechano-regulation model by Prendergast et al. [22], determined the optimal dimensions that the pores of scaffolds 3D printed with the FDM technique and including spherical pores, respectively, must possess. In this model, the fracture site is modelled as a biphasic poroelastic material, and the biophysical stimulus that triggers the osteogenic differentiation of the mesenchymal stem cells is hypothesized to be a function of the octahedral shear strain and of the interstitial fluid flow measured in the regenerating tissue. The authors, by using this model, defined, via an optimization algorithm, the optimal dimensions of pores for different load values acting on the scaffold [10,17]. Martinez-Marquez et al. [18], in their review paper, used the quality by design system to explore the quality target product profile and ideal quality attributes of additively manufactured titanium porous scaffolds for bone regeneration with a biomimetic approach. The systematic literature review presented an overview of the reported properties in research studies of fully porous titanium bone implants fabricated with additive manufacturing published in the last two decades. Unit cell geometry, porosity, elastic modulus, compressive yield strength, ultimate compressive yield strength, and compressive fatigue strength were systematically reviewed and benchmarked against the proposed ideal quality attributes.

The studies dealing with applicative/clinical aspects investigate very wide and interesting topics. The effects of chronic alcoholism on the repair of bone defects associated with xenograft was investigated by German et al. [21]. The interesting review paper by Stokovic et al. [19] summarizes the bone regeneration strategies and the animal models used for the initial, intermediate, and advanced evaluation of promising therapeutical solutions for new bone formation and repair. Dentistry issues were investigated by Grassi et al. [20], who evaluated the clinical success of horizontal ridge augmentation in severely atrophic maxilla using freeze-dried, custom-made bone harvested from the tibial hemiplateau of cadaver donors.

All the papers of the Special Issue were submitted to peer review, and thanks to the help of the reviewers, the quality of all the manuscripts was significantly improved. My special thanks go, therefore, to the authors for their excellent contribution, to the reviewers, for their invaluable help, as well as to the editorial staff of *Materials*, in particular to Ariel Zhou, Section Managing Editor for her kind assistance, competence and patience.
